# Targeting Syndecan-1, a molecule implicated in the process of vasculogenic mimicry, enhances the therapeutic efficacy of the L19-IL2 immunocytokine in human melanoma xenografts

**DOI:** 10.18632/oncotarget.6055

**Published:** 2015-10-09

**Authors:** Paola Orecchia, Romana Conte, Enrica Balza, Gabriella Pietra, Maria Cristina Mingari, Barbara Carnemolla

**Affiliations:** ^1^ Laboratory of Immunology, IRCCS AOU San Martino-IST, Genoa, Italy; ^2^ Laboratory of Cell Biology, IRCCS AOU San Martino-IST, Genoa, Italy; ^3^ Department of Experimental Medicine, University of Genoa, Genoa, Italy

**Keywords:** vasculogenic/vascular mimicry, angiogenesis, scFv OC-46F2 anti Syndecan-1, melanoma combined therapy, immunocytokine L19-IL2

## Abstract

Anti-angiogenic therapy of solid tumors has until now failed to produce the long lasting clinical benefits desired, possibly due to the complexity of the neoangiogenic process. Indeed, a prominent role is played by “vasculogenic” or “vascular” mimicry (VM), a phenomenon in which aggressive cancer cells form an alternative microvascular circulation, independently of endothelial cell angiogenesis. In this study we observed, in melanoma patient cell lines having vasculogenic/stem-cell like phenotype and in melanoma tumors, the syndecan-1 co-expression with VM markers, such as CD144 and VEGFR-2. We show that melanoma cells lose their ability to form tubule-like structures *in vitro* after blocking syndecan-1 activity by the specific human recombinant antibody, OC-46F2. Moreover, in a human melanoma xenograft model, the combined therapy using OC-46F2 and L19-IL2, an immunocytokine specific for the tumor angiogenic-associated B-fibronectin isoform(B-FN), led to a complete inhibition of tumor growth until day 90 from tumor implantation in 71% of treated mice, with statistically significant differences compared to groups treated with OC-46F2 or L19-IL2 as monotherapy. Furthermore, in the tumors recovered from mice treated with OC-46F2 either as monotherapy or in combination with L19-IL2, we observed a dramatic decrease of vascular density and loss of VM structures. These findings indicate for the first time a role of syndecan-1 in melanoma VM and that targeting syndecan-1, together with B-FN, could be promising in improving the treatment of metastatic melanoma.

## INTRODUCTION

Building on the premise that blood vessels are essential for tumor growth and metastasis in all solid tumors, including melanoma, anti-angiogenic therapy was originally developed to “starve” primary and metastatic tumors by blocking blood vessel recruitment [1-6]. However, while anti-angiogenic drugs are effective at reducing angiogenesis, these therapies have not produced widespread or long lasting clinical benefits and have shown their limitations, due to many other mechanisms involved in tumor progression and regulated by the tumor microenvironment [3, 7-9].

Metastatic melanoma is one of the deadliest forms of cancer; progress in its treatment, however, is very limited [10-13]. Recently L19-IL2, an immunocytokine specific for the angiogenesis-associated B-fibronectin isoform able to selectively accumulate on tumor neovasculature, was investigated in a randomized phase II clinical trial in patients with metastatic melanoma in combination with dacarbazine or with L19-TNFα. Thus far, L19-IL2 has yielded encouraging results [10, 14-18].

It is known that in many malignant tumors, including metastatic melanoma, other forms of blood supply in tumor tissues exist in addition to angiogenesis. One of these mechanisms is “vasculogenic” or “vascular” mimicry (VM), in which cells of a highly aggressive malignant tumor can form vascular-like channels without implicating endothelial cells, thereby providing nutrients for tumor growth [19, 20]. An emerging premise is that, in the cure of solid tumors, the most effective therapies will entail targeting combinations of factors for drug delivery. Markers of VM may therefore be important targets for this purpose [7-9, 20].

Many adhesion and membrane proteins, including MMP1/2, VEGFR-1/2, HIF-1, Nodal, FAK, EpHA2 and VE-cadherin (CD144), seem to be crucial to VM formation in many types of solid tumors such as melanoma and glioma [20-24]. VE-cadherin, specifically expressed in endothelial cells, is also expressed in aggressive melanoma cells and its expression knockdown inhibits VM formation, thus making it a major candidate marker of VM [25]. Recent studies have also implicated some cancer stem cell (CSC) markers such as CD133, ALDH1 and CD44 in VM formation [20].

Syndecan-1 (CD138), one of the four members of the syndecan family, is a cell surface heparan (HS) and chondroitin sulphate (CS) proteoglycan. Syndecan-1 is expressed predominantly in epithelial cells [26], but it is also found in B lymphocytes at specific stages of differentiation [27]. More recently, it was shown to be expressed also in malignant melanoma cells [22]. Syndecan-1 expression correlates to increased metastatic potential in melanoma cells [28]. The structural features of the HS-chains are responsible for the interaction of Syndecan-1 with a number of soluble factors, including pro-angiogenic factors like Vascular Endothelial Growth Factor (VEGF) and Fibroblast Growth Factor-2 (FGF-2), cell-associated molecules and extracellular matrix (ECM) components [26, 29, 30]. Furthermore, elevated levels of VEGF and shed Syndecan-1 form matrix-anchored complexes that together activate integrin and VEGF receptors on endothelial cells, thereby stimulating tumor angiogenesis [31]. A number of findings suggest that Syndecan-1 is involved in the stimulation of CSC or tumor initiating cells (TIC) and that this can affect disease relapse and resistance to therapy [30] (and refs therein).

We recently demonstrated that Syndecan-1 is a potential therapeutic target in melanoma and ovarian carcinoma. In fact, preclinical experiments of therapy in subcutaneous graft models of melanoma and ovarian carcinoma showed that scFv OC-46F2, specific for the ectodomain of Syndecan-1, inhibited vascular maturation and tumor growth [22].

In this study we demonstrate by *in vitro* and *in vivo* experiments that OC-46F2 antibody was able to inhibit the vascular mimicry of melanoma cells and vascular structure formation of endothelial cells. These findings indicate, for the first time, that Syndecan-1 is implicated in the process of vascular mimicry in melanoma. We report that OC-46F2, administered systemically in combination with L19-IL2, leads to a complete inhibition of tumor growth until day 90 from tumor implantation in 71% of treated mice. Moreover, at day 124 in the L19-IL2/OC-46F2 group, the tumor free survival was 64% in contrast to 0% observed in the L19-IL2 treated group. These results suggest that the combined therapy could improve the therapeutic efficacy of both OC-46F2 and L19-IL2 administered as single agents.

## RESULTS

### Characterization of human metastatic melanoma cells showing vasculogenic phenotype

We tested melanoma cell lines SKMEL28, MV3 and melanoma cells isolated from ten patients, all positive for Syndecan-1, to form tubule-like structures on Matrigel. Moreover, the ability of all cell lines to induce tumor growth and lung metastasis when injected subcutaneously or in the tail vein of NOD SCID mice, respectively, was assessed. As summarized in Table 1, SKMEL28, MV3, MeTA and MeMO were able to form tubule-like structures on Matrigel, and six out of seven subcutaneously inoculated melanoma cells isolated from patients were able to induce tumor growth as SKMEL28 cell line. Moreover, SKMEL28 and the two cell lines MeTA and MePA were able to metastatize to the lung after i.v. injections, as already described for the metastatic cell line MV3 [32]. To detect the human metastatic nodules we stained lung sections with the anti human Ki67 antibody that specifically recognizes human cells in proliferation (Supplementary Figure S1 A). Furthermore, we analyzed the c-Kit (CD117) expression and, in accordance with the literature [33], we observed that melanoma cells with a strong metastatic potential, such as SKMEL28, MePA, MeTA and MV3, were negative for c-Kit expression, in contrast to MeMI that expressed c-Kit (Table 1, Supplementary Figure S1 B and S2) and was unable to form metastases. We analyzed all melanoma cell lines for their expression of melanoma stem cell markers CD133/1 and CD271 by cytofluorimetric analysis. While CD133/1 was expressed only on MeTA, the majority of melanoma cell lines with vasculogenic phenotype were positive with CD271 (Supplementary Figure S2). Moreover, all melanoma cell lines expressed as mRNA other markers of cancer stem cells, such as CD44, ALDH1 and Nodal (data not shown).

**Table 1:** Human metastatic melanoma cells characteristics associated to VM

Cell lines	vasculogenic phenotype	human tumors xenografts/NOD SCID mice	lung metastasis/NOD SCID mice	c-Kit expression
**SKMEL28**	yes	yes	yes	no
**MV3**	yes	nd	yes	no
**MeTA**	yes	yes	yes	no
**MeMO**	yes	no	nd	no
**MeMI**	no	yes	no	yes
**MePA**	no	yes	yes	no
**MeOV**	no	yes	nd	no
**MeCoP**	no	yes	nd	yes
**MeFeR**	no	yes	nd	yes
**MeBO**	no	nd	nd	yes
**MeTU**	no	nd	nd	yes
**MeDeBO**	no	nd	nd	yes

Abbreviations: nd, not determined

**Figure 1:**
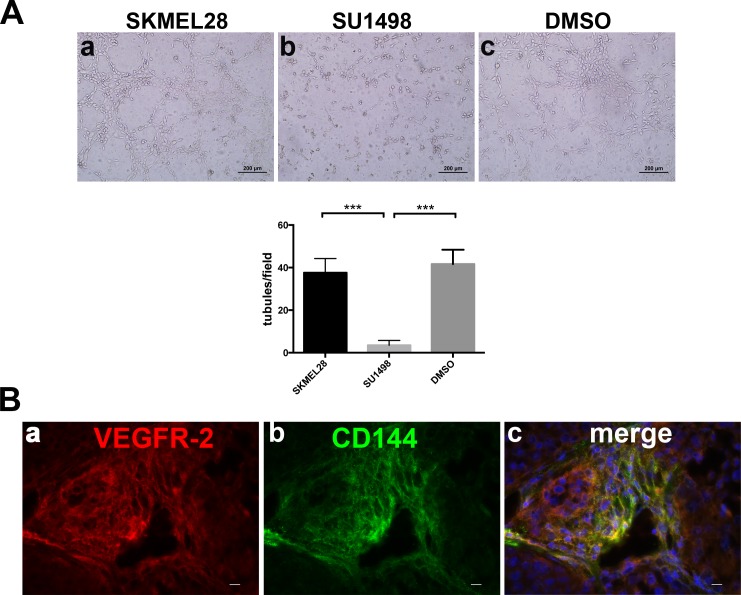
VEGFR-2 is involved in melanoma VM **A.**, *In vitro* Matrigel tube formation using melanoma cells SKMEL28 in presence of SU1498 (b) compared to untreated (a) or DMSO (c) treated cells. The differences in tubule formation were quantified by column bar graphs reported below the experiments. *** indicates extremely significant differences between treated and DMSO or untreated cells. Scale bars, 200 μm. The mean ± SEM are indicated. **B.**, representative immunofluorescence of cryostat sections of SKMEL28/NOD SCID, stained with anti VEGFR-2 (a) and anti CD144 (b). Merged image shows co-localization of VEGFR-2 with CD144 (c). Scale bars, 10 μm.

We previously reported in Orecchia et al. 2013, that VEGFR-2 co-localized with Syndecan-1 in human melanoma xenograft. To investigate the role of VEGFR-2 in the melanoma VM, we performed *in vitro* Matrigel experiments with or without SU1498, a specific VEGFR-2 kinase inhibitor using melanoma cells. As shown in Figure 1A, SU1498 inhibits the *in vitro* formation of tubule-like structures (b) compared to treated with DMSO (c) or not treated (a) cells. Moreover, by immunofluorescence staining on SKMEL28/NOD SCID sections, we show that VEGFR-2 (Figure 1B, a) co-localizes with CD144 (Figure 1B, b).

**Figure 2:**
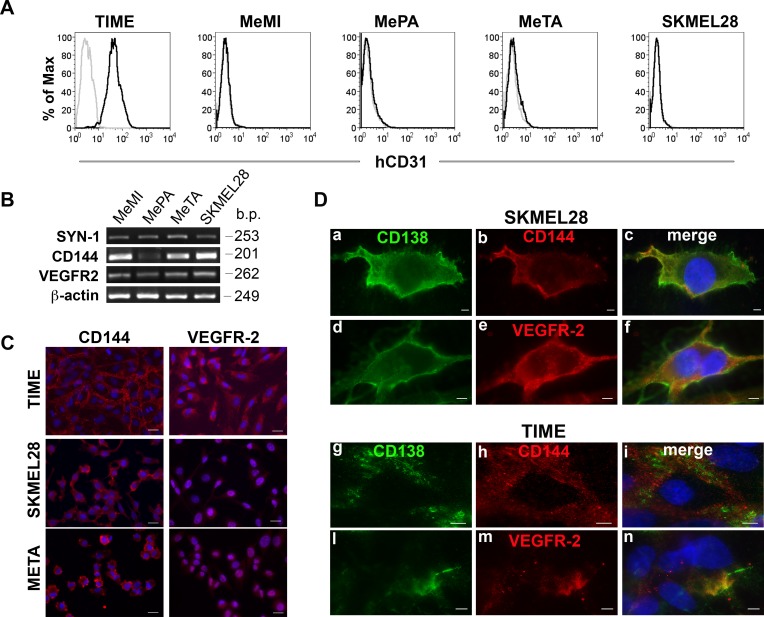
Expression of human CD31, CD144 and VEGFR-2 in positive Syndecan-1 melanoma cells **A.**, Flow cytofluorimetric analysis of human CD31 expression in TIME endothelial cell and MeMI, MePA, MeTA and SKMEL28 human melanoma cells. Gray profiles represent negative controls. **B.**, RT-PCR analysis of human Syndecan-1, CD144 and VEGFR-2 expression in MeMI, MePA, MeTA and SKMEL28 human melanoma cells. Beta-actin was used as positive control. Base pairs (b.p.) of human genes are indicated. **C.**, Immunofluorescence analysis of TIME endothelial cells, SKMEL28 and MeTA human melanoma cells stained with anti-CD144 or anti VEGFR-2 counterstained with DAPI. Scale bars, 20 μm. **D.**, Immunofluorescence analysis of SKMEL28 human melanoma cells and TIME endothelial cells in which co-distribution of syndecan-1 (CD138) with CD144 or VEGFR-2 is shown. The merged signals are shown in the right panel (c, f, i, n). Scale bars, 10 μm.

We chose three representative melanoma cells from patients (MeMI, MePA and MeTA) and SKMEL28 cell line and we observed that they were negative for the expression of human CD31, a marker of endothelial cells (Figure 2A and Supplementary Table S1), but that they expressed CD144 and VEGFR-2 (Figure 2B, 2C and Supplementary Table S1). These results indicate that tubular-like structures formed by melanoma tumor cells were not of endothelial origin. In Figure 2D, using OC-46F2, we show representative micrographs in which Syndecan-1 (CD138) co-localized with CD144 (a-c; g-i) and VEGFR-2 (d-f; l-n) in SKMEL28 and TIME cells.

**Figure 3:**
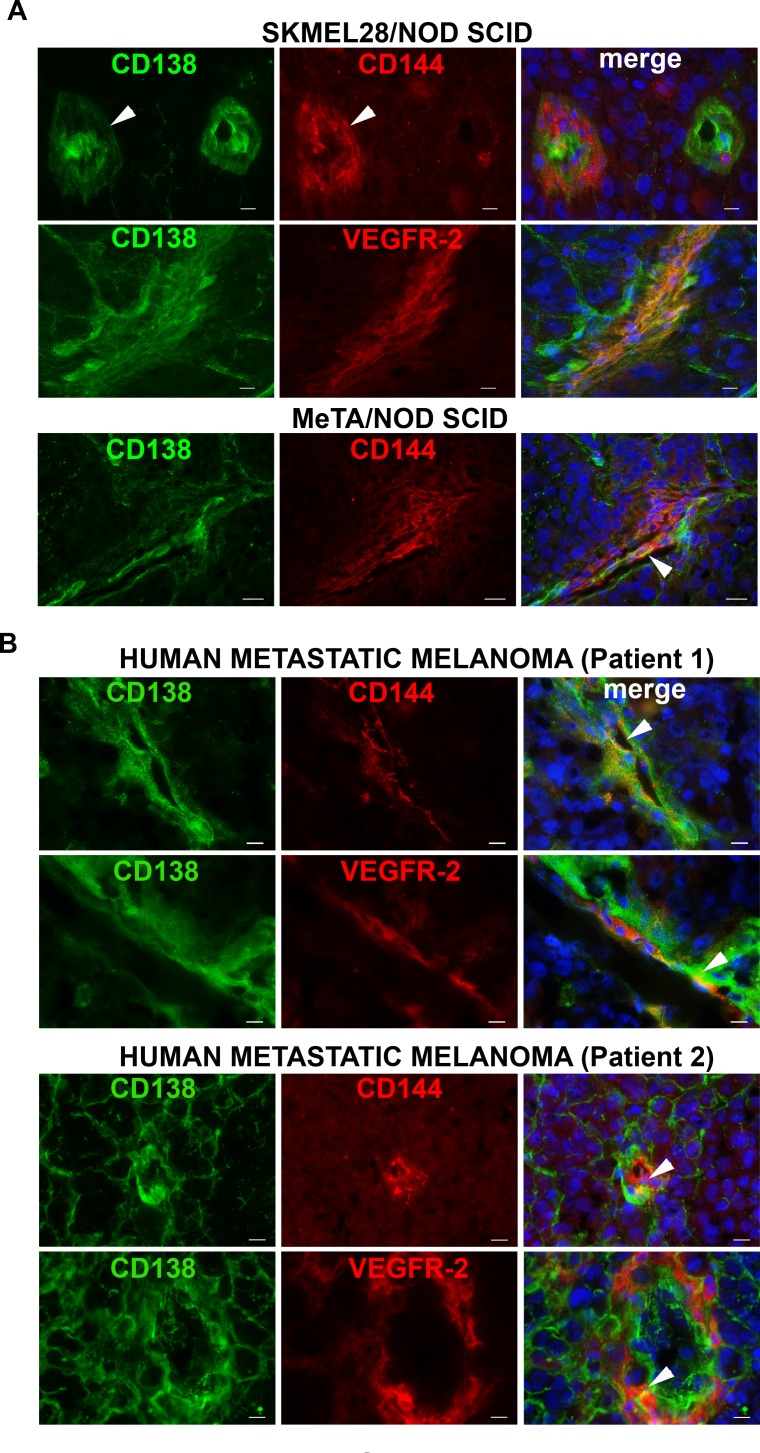
Syndecan 1 co-localizes with CD144 and VEGFR-2 in melanoma tissues Immunofluorescence analysis of cryostat sections of SKMEL28 and MeTA melanoma tumors in NOD SCID mice **A.** and of human metastatic melanoma from two patients **B.** double stained with OC-46F2 and anti-VEGFR-2 or anti-CD144 antibodies as indicated in each picture and counterstained with DAPI. The merged signals are shown in the right panel. Scale bars, 10 μm.

In Figure 3A, by immunofluorescence staining on SKMEL28/NOD sections, we show that OC-46F2 was able to recognize vessels of human origin. In fact, the same vessels were positive with CD144 antibody specific for the human protein as indicated by arrowheads. Moreover, we show that Syndecan-1 co-localizes with VEGFR-2. The same results were observed with staining on MeTA/NOD SCID sections. In human metastatic melanoma biopsies obtained from different patients (Figure 3B) the same co-localization of Syndecan-1 with CD144 or VEGFR-2 was observed. All these results suggest that Syndecan-1 is present in the same tubular like structures expressing CD144 and VEGFR-2 markers of VM.

### Human Syndecan-1, CD144 and VEGFR-2 expression in experimental lung metastasis of human melanoma

**Figure 4:**
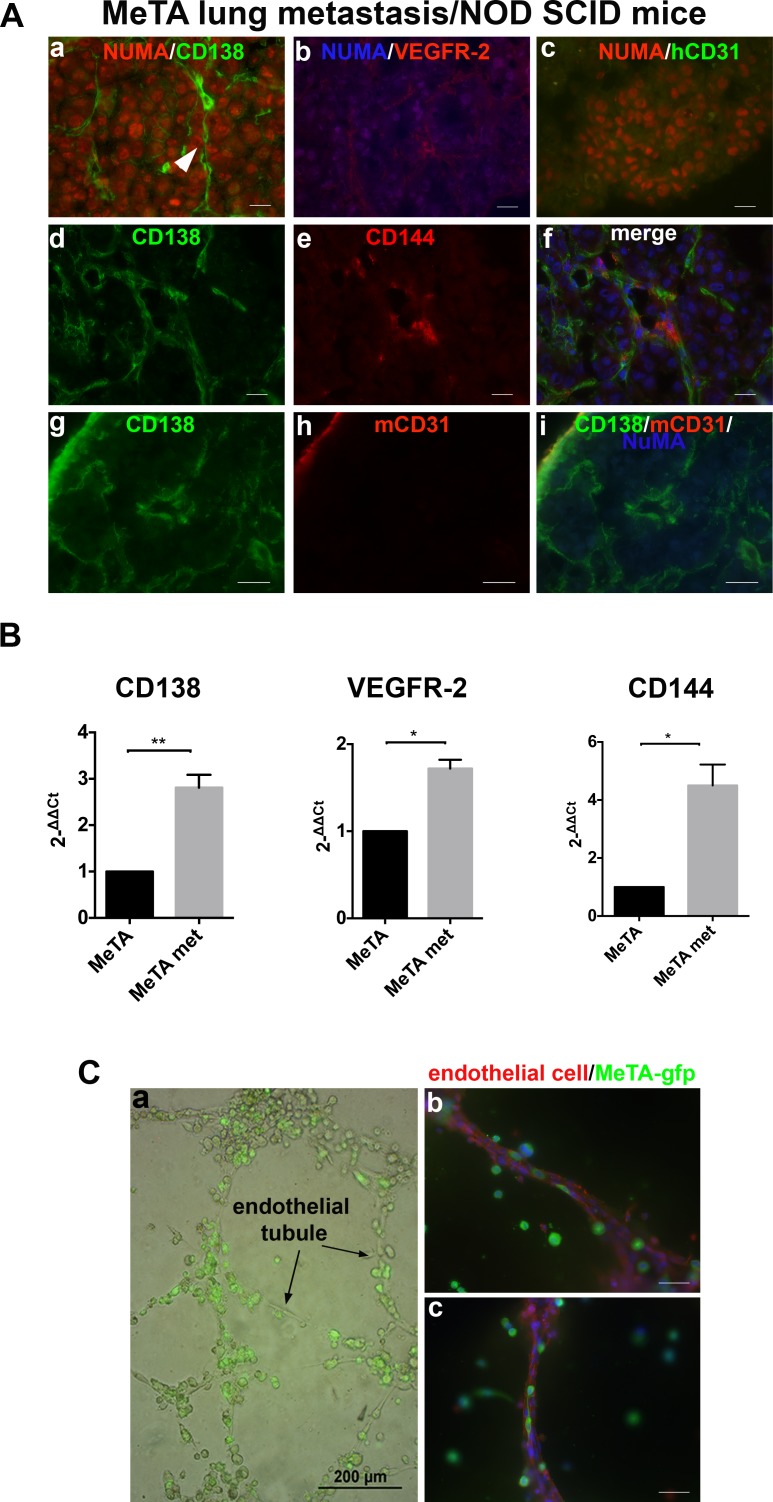
Expression of Syndecan-1 in lung metastases **A.**, Immunofluorescence of cryostat sections of lung metastasis induced by i.v. MeTA injection in NOD SCID mice double stained with anti-human NuMA and OC-46F2, anti-VEGFR-2, anti-hCD31, anti-hCD144 and anti mouse CD31 antibodies as indicated in each picture. Panel A (d-f) is counterstained with DAPI. Scale bars, 10 μm. **B.**, qRT-PCR analysis of human Syndecan-1, VEGFR-2 and CD144 expression in human melanoma metastatic cells MeTA met in comparison to MeTA. 2-[Δ][Δ]Ct is reported in the column bar graph. *, **, indicate significant and very significant differences, between the two types of cells. **C.**, Real time image of MeTA-GFP migration along endothelial tubules. Scale bars, 200 μm (a). Fluorescent staining of tubular structures growth on Matrigel using anti-GFP antibody to localize melanoma cells and anti-human CD31 antibody specific for endothelial cells (b-c). Scale bars, 50 μm.

To investigate the involvement of human Syndecan-1 in VM during the metastatic process, we performed immunofluorescence staining on lung sections of MeTA intravenously injected in NOD SCID mice. To specifically recognize all human melanoma cells, we used anti-human NuMA antibody and we observed a strong expression of Syndecan-1 (Figure 4A, a) and VEGFR-2 (Figure 4A, b) in NuMA positive melanoma cells. These data correlate with the qRT-PCR results, in which we observed a significant increase of Syndecan-1 mRNA expression in MeTA human melanoma cells isolated from murine lung metastases (MeTA met) compared to injected cells, as seen for VEGFR-2 and CD144 (Figure 4B). In Figure 4A we show, using OC-46F2 and anti human CD144 antibody (d-f), the presence of Syndecan-1 in tubule-like structures of human origin. All lung metastasis specimens were negative for human CD31 (c). Furthermore, the human tubule-like structures positive for OC-46F2 were negative for mouse CD31 (g-i). Figure 4C shows real time images of MeTA-GFP migration along endothelial tubules (a), and fluorescent staining, using anti-GFP and anti-human CD31 antibodies, illustrates the capacity of these tumor cells to localize along the external surface of vascular tubules, revealing a tropism for the vascular channels (b, c).

### Anti Syndecan-1 human recombinant antibody, OC-46F2, inhibits vascular mimicry and angiogenesis in *in vitro* and *in vivo* Matrigel experiments

To evaluate the capacity of OC-46F2 to inhibit vascular mimicry and angiogenesis, we performed *in vitro* experiments on Matrigel using SKMEL28, MeTA and TIME.

**Figure 5:**
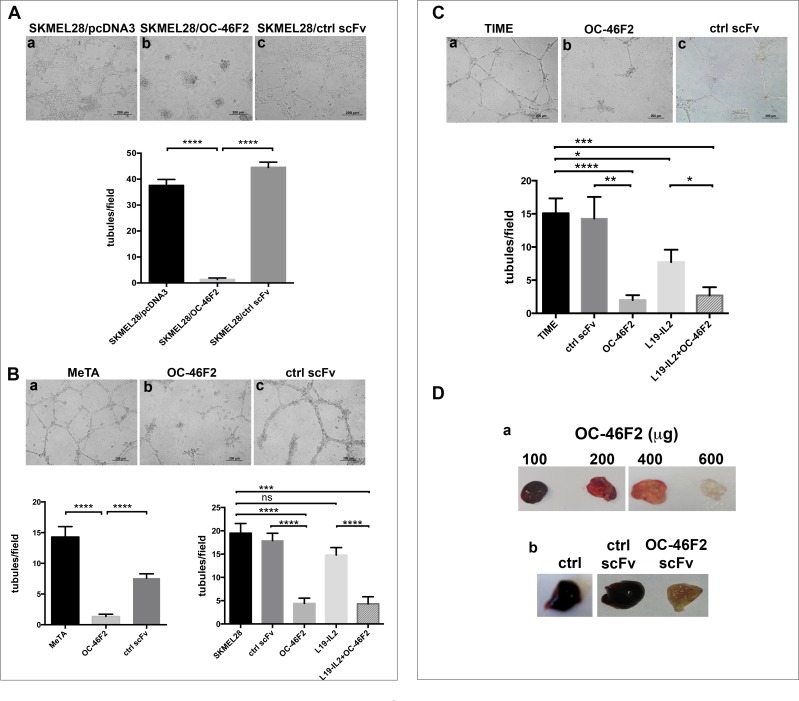
OC-46F2 inhibits vascular mimicry and angiogenesis **A.**, SKMEL28 stably transfected cells with OC-46F2 were not able to form tubule-like structures after 48 hours from plated cells on Matrigel (b) in comparison to cells transfected with the empty vector (a) or with a control scFv (c) that formed well organized structures. **B.**, *in vitro* Matrigel tube formation using MeTA melanoma cells (a-c) or SKMEL28 in presence of OC-46F2 and/or L19-IL2 in comparison to control scFv treated or untreated cells. **C.**, *in vitro* Matrigel vascular channel formation using TIME endothelial cells (a-c) in presence of OC-46F2 and/or L19-IL2 in comparison to control scFv treated or untreated cells. The differences in tubule formation at different treatments were quantified by column bar graphs reported below the corresponding experiments. Symbols indicate statistically differences between the groups connected by lines: ns, not significant; *, significant; **, very significant and ***, **** extremely significant. Scale bars, 200 μm. The mean ± SEM are indicated. **D.**, Photographs of plugs removed from OC-46F2 treated mice at different dose (a) and of representative plugs taken from untreated (*n* = 6), control scFv (*n* = 6) or OC-46F2 treated mice (*n* = 6) (b).

As shown in Figure 5A, SKMEL28 melanoma cells stably transfected with scFv OC-46F2 were not able to form tubule-like structures after 48 hours plated cells on Matrigel (b) in comparison to cells transfected with the empty vector (a) or with a control scFv (c) that formed well organized structures. A similar inhibitory effect of OC-46F2 was obtained by adding the antibody at the concentration of 200 μg/ml to the cells. In fact, the *in vitro* formation of tubule-like structures by SKMEL28 and MeTA melanoma cells (Figure 5B) and of vascular structures by TIME endothelial cells (Figure 5C) was inhibited by scFv OC-46F2 (Figure 5B, b; 5C, b) compared to cells treated with control scFv (Figure 5B, c; 5C, c) or not treated (Figure 5B, a; 5C, a). No inhibition or a slight reduction of vascular channel formation was observed by adding L19-IL2 to SKMEL28 melanoma cell line (Figure 5B, right column bar graph) or TIME endothelial cells (Figure 5C), respectively. Moreover, we evaluated the anti-angiogenic effect of OC-46F2 using *in vivo* Matrigel plug assay. Indications of an anti-angiogenic effect in response to different doses of OC-46F2 can be assessed visually because of the color difference in the plugs (Figure 5D, a). As shown in Figure 5D, b, the representative plug removed from OC-46F2 treated mice showed no FGF2-induced vascularization in contrast to plugs taken from control scFv treated or untreated mice.

These results suggest that Syndecan-1 is involved in the tubule-like formation of melanoma cells and vascular structure formation of endothelial cells. Furthermore, since OC-46F2 recognizes an epitope of the core protein of Syndecan-1, as demonstrated in Supplementary Figure S3 by treating human melanoma cells with PNPX, an elongation inhibitor of protein-associated glycosaminoglycan (GAG), it is conceivable that GAG chains of the molecule may not be implicated in these mechanisms.

### Therapeutic efficacy of OC-46F2 in combination with L19-IL2 against human melanoma xenograft

**Figure 6:**
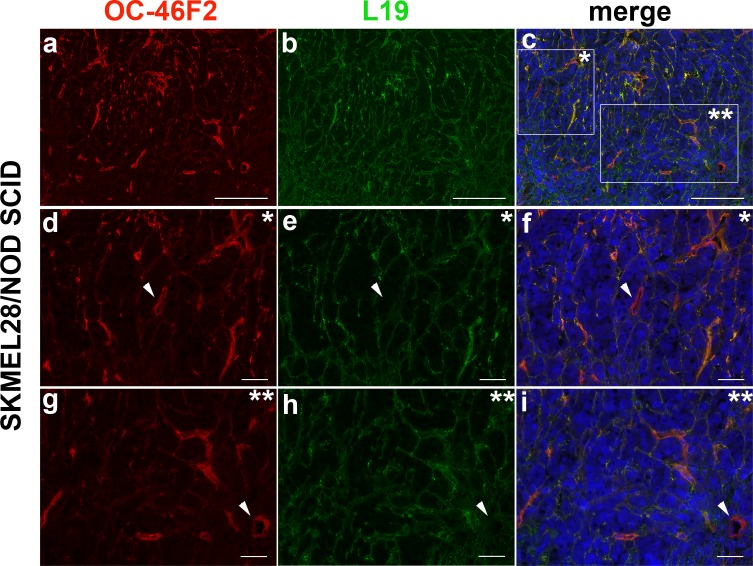
Immunofluorescence of cryostat sections of SKMEL28 melanoma tumors in NOD SCID mice double stained with OC-46F2 (a) and L19 (b) antibodies counterstained with DAPI The merged signals show co-distribution of Syndecan-1 with B-fibronectin in some vascular structures (c); in * and ** magnifications, some vessels were positive with OC-46F2 only, as indicated by white arrowhead (d-f and g-i). Scale bars, 200 μm (a-c) or 50 μm (d-i).

We recently reported that the human recombinant antibody, scFv OC-46F2, specific for the ectodomain of Syndecan-1, exerts an antitumor activity *in vivo* by reducing tumor growth and vascular maturation in experimental human melanoma and ovarian carcinoma murine models [22]. In line with our already published data [15, 22] we confirmed that scFv OC-46F2 and L19, an scFv specific for the angiogenesis-associated B-fibronectin isoform, stain both ECM and tumor vessels (Supplementary Figure S4). Interestingly, in this study we observed that the anti Syndecan-1 antibody, OC-46F2, stained some intratumoral vessels that were negative with L19 antibody. In fact, as shown in the representative Figure 6 (a-c) and in its magnifications (d-f, g-i), both OC-46F2 and L19 stain the majority of the vessels in the experimental human melanoma SKMEL28; however some vessels were positive with OC-46F2 only (see arrowheads in Figure 6, d-f, g-i).

To investigate if a combined therapy with another drug, able to selectively localize to the tumor structures, could enhance the antitumor effect of scFv OC-46F2, we chose L19-IL2, an immunocytokine composed of human scFv L19 and IL2 [16, 34, 35].

**Figure 7:**
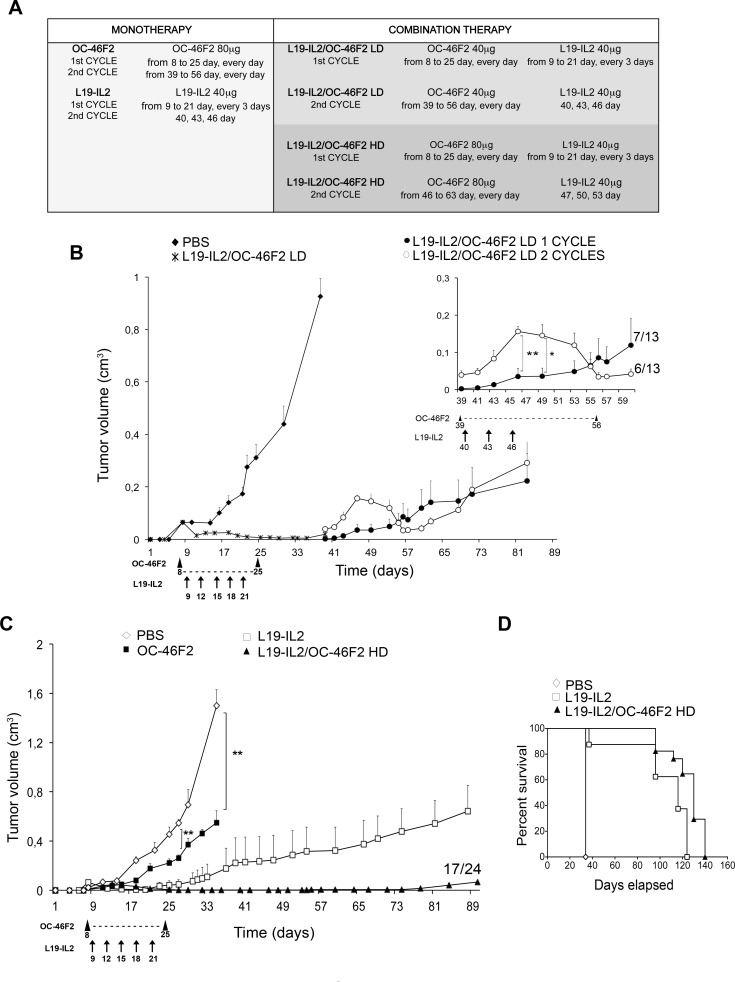
Targeting Syndecan-1 by scFv OC-46F2 enhances the therapeutic efficacy of immunocytokine L19-IL2 in melanoma **A.**, Administration schedule of OC-46F2 and L19-IL2 as single or combined treatments. Reported days are from tumor cells s.c. implantation. LD, Low Dose; HD, High Dose. Combination therapy experiments were started eight days after SKMEL28 human melanoma implantation in NOD SCID mice, when palpable tumors had developed. Mice were randomized in the different treatment groups. **B.**, Tumor growth inhibition in mice (n=13) treated with L19-IL2 and OC-46F2 low dose administered in combination for eighteen days. In the upper right side a magnification of growth curve from day 39 is reported. Only 40% of mice (*n* = 6) were subjected to a second cycle of combination therapy of OC-46F2 every day for eight days and L19-IL2 on days 40, 43, 46 as indicated by the arrows. * and ** indicate significant and very significant differences, respectively, between the group treated with second cycle and untreated group at days 46 and 49. **C.**, Tumor growth inhibition in mice (n=24) treated with L19-IL2 and OC-46F2 at high dose administered as monotherapy or in combination. ** indicates very significant difference between the OC-46F2 treated and untreated groups at days 27 and 35. From day 32 the two-tailed p value of L19-IL2/OC-46F2 high dose group of mice compared to OC-46F2 and L19-IL2 groups was extremely significant (*p* < 0.0001, ****) and very significant (*p* = 0.0037, **), respectively. The mean tumor volumes ± SEM are indicated. Statistical significance of the differences between the groups was evaluated by nonparametric Mann Whitney test. **D.**, Tumor free survival curves versus time (days) of SKMEL28 melanoma bearing mice treated with L19-IL2 as monotherapy, L19-IL2 and OC-46F2 HD as combination therapy or untreated. Arrowheads indicate the OC-46F2 treatment administrated every day in the reported range of days. Arrows indicate the L19-IL2 treatment administrated in the reported days.

Combination therapy experiments were performed in NOD SCID mice bearing SKMEL28 human melanoma. In a preliminary experiment, the doses used were 40 μg for OC-46F2 and 40 μg for L19-IL2 immunocytokine (given in combination) for each administration. The combination schedule of therapy is described in Materials and Methods and summarized in Figure 7A. As shown in Figure 7B until day 35 from melanoma cell subcutaneous implantation we observed a drastic reduction of tumor growth in 100% of mice compared to the untreated control group. From day 39, six out of thirteen mice (40%) presented an increase of tumor volume; therefore a second cycle of combined therapy was administered following the combination schedule reported in Figure 7A. As shown in the upper right side of the Figure 7B, in all six treated mice the tumor volumes decreased from day 49 to day 56 when mice received OC-46F2 only. The remaining seven out of thirteen mice (60%) received no treatment. The two-tailed p value of the two groups at day 46 and 49 from tumor implantation was very significant (*p* value = 0.0058) and significant (*p* value = 0,0192), respectively. In this preliminary experiment the group of mice treated with the combined therapy shows a delay of 60 days of tumor growth respect to the control group that did not receive any therapy. A second experiment was carried out using a larger number of mice (*n* = 24) in order to have three groups that would receive a second cycle of therapy as follows: L19-IL2 and OC-46F2 as single therapy or in combination. Moreover, because OC-46F2 is completely non-toxic, we increased the dose of OC-46F2 to 80 μg (high dose, HD), leaving the dose of L19-IL2 unchanged due to its toxicity. As shown in Figure 7C groups of mice were untreated or treated with OC-46F2, L19-IL2 as single therapy or combination therapy. The combination therapy (HD) completely inhibited tumor growth until day 90 in seventeen out of twenty four treated mice (71%). From day 32 the two-tailed p value of L19-IL2/OC-46F2 high dose group compared to OC-46F2 and L19-IL2 groups of mice was extremely significant (*p* < 0.0001) and very significant (*p* = 0.0037), respectively, indicating the strong efficacy of combined therapy (Figure 7C). In fact, at day 124 in the L19-IL2/OC-46F2 high dose group, the tumor free survival was 64% compared to 0% in L19-IL2 treated group (Figure 7D). To microscopically analyze tumor sections following combination treatments, the experiment was interrupted at day 140 when the tumor volumes were between 0.8 and 1.6 cm^3^. None of the groups exhibited a body weight loss greater than 3% at any time point during the treatments. The seven not completely responding mice out of twenty four (29%) were randomized into three groups and subjected to a second cycle of therapy, as reported previously, following the treatment schedule described in Figure 7A for the mono- and combined therapy. A delay in tumor growth was observed in the group in which the two molecules were administered together (Supplementary Figure S5).

**Figure 8:**
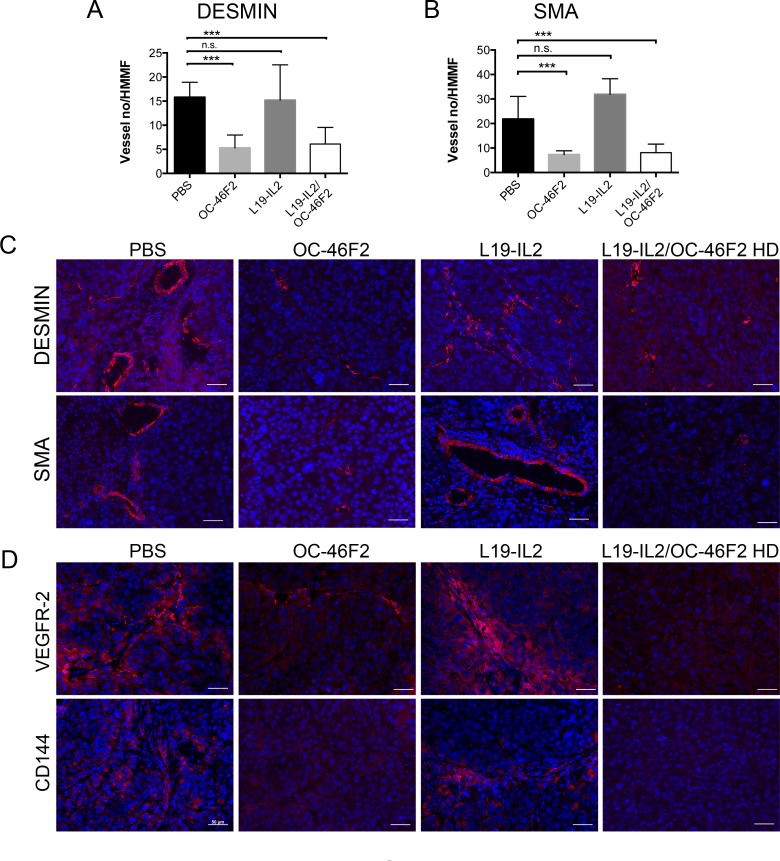
Therapeutic treatment with OC-46F2 inhibits vascular maturation and induces loss of VM structures Quantification of **A.** immature blood vessels (Desmin staining) and **B.** mature blood vessels (SMA staining) per HMMF in SKMEL-28 tumors subjected to different types of treatments. The mean ± SEM are indicated. *** indicates extremely significant differences between OC-46F2 or L19-IL2/OC-46F2 HD treated and untreated groups. n.s. indicates not significant differences between L19-IL2 treated and untreated groups. Immunofluorescence analysis of cryostat sections of tumors recovered from SKMEL28/NOD SCID mice subjected to the different type of treatments stained with anti-desmin or anti-SMA **C.** and anti-VEGFR-2 or anti-CD144 **D.** antibodies as indicated in each picture and counterstained with DAPI. Scale bars, 50 μm.

Figure 8A-8C shows a drastic reduction of vessel numbers in tumors from mice that received OC-46F2 either as monotherapy or combination therapy, as revealed using anti-Desmin and anti-SMA antibodies, markers of early and mature vessels, respectively. Moreover, the expression of the two VM markers, VEGFR-2 and CD144, was almost negative in the tumors from animals receiving OC-46F2 therapy (Figure 8D). These *in vivo* experiments evaluating the change in vascular density and VM structures in the tumor tissues taken from treated mice confirm the efficacy of OC-46F2 in VM inhibition as above reported in *in vitro* results.

## DISCUSSION

Poor five-year survival has been observed in some VM-forming aggressive cancers, including melanoma. Furthermore, a higher rate of VM has been reported in melanoma patients with metastasis compared to patients with primary melanoma, indicating that VM is able to promote tumor metastasis [20, 36]. Recently accumulating research focuses on the availability of new anticancer treatments able to inhibit the formation of VM that could be combined with anti-angiogenic therapies. Thalidomide, one of the few anti-angiogenic agents that had shown efficacy in suppressing VM channels and mosaic vessel formation in an experimental model of melanoma, was never used in clinical applications due to its severe teratogenic effects [37]. Moreover, some anti-angiogenic agents, such as the monoclonal antibody bevacizumab, or endostatin and TNP-470, had no effect on VM; others, such as sunitinib, accelerate tumor cell VM in triple-negative breast cancer [23, 38-40]. The spectrum of therapeutic options for the cure of patients with advanced melanoma continues to expand, and a new anti-angiogenic drug, the immunocytokine L19-IL2, has been used in experimental phase II clinical trials for this deadly form of cancer [10, 14-18].

We recently reported that blocking Syndecan-1 activity *via* the human specific antibody scFv OC-46F2 leads to an antitumor effect by inhibiting vascular maturation and tumor growth in experimental human melanoma and ovarian carcinoma models [22]. In this study we observed that VM of melanoma cells was inhibited by SU1498, a specific VEGFR-2 kinase inhibitor, as demonstrated in glioblastoma cell lines [23]. Our results are consistent with our previously reported data and with many reports which describe an interaction between Syndecan-1 and VEGFR-2 [22, 23, 41, 42]. However, other VEGF receptors could be involved in VM, as recently reported for VEGFR-1 [24]. We demonstrated that Syndecan-1 positive melanoma cell lines having a vasculogenic and a stem cell-like phenotype, express two molecules involved in melanoma VM, such as CD144 and VEGFR-2 [20, 43]. Moreover, all these cell lines are negative with anti-CD31, differently from Dunleavey et al., who described a subpopulation of melanoma cells positive for CD31 but negative for VEGFR-2 [44]. We observed an increased Syndecan-1 mRNA expression in human melanoma cells isolated from murine lung metastases (MeTA met) compared to the injected cells (MeTA). Analyzing lung metastatic tissues we have shown that Syndecan-1 is expressed in the early stage of metastatic development, similarly to VEGFR-2 and CD144. Furthermore, the *in vitro* and *in vivo* experiments have shown that the antibody OC-46F2 was able to inhibit human melanoma VM and to block the pro-angiogenic activity of Syndecan-1.

In a comparative study of Syndecan-1 and angiogenesis-associated B-fibronectin isoform expression in melanoma tissues, using OC-46F2 and L19 antibodies, respectively, we observed that some vessels were positive with the anti-Syndecan-1 antibody but negative with L19. These preliminary observations afforded the possibility to investigate the effectiveness of OC-46F2 in combination with the L19-IL2 immunocytokine in preclinical therapeutic experiments. Furthermore, the use of a human melanoma cell line in immune-compromised NOD SCID mice allowed us to study the human or murine origin of tumor vessels and to verify the efficacy of the two treatments specific for different targets of tumor stroma.

We found that the combination therapy using the OC-46F2 anti Syndecan-1 blocking antibody and the immunocytokine L19-IL2 leads to a complete inhibition of tumor melanoma growth until day 90 from tumor implantation in 71% of treated mice. A second cycle of combined therapy in the not completely responding mice was efficacious to induce a further delay in tumor growth. Moreover, by monitoring mice for a longer time, we observed that at day 124, the tumor free survival was 64% in the L19-IL2/OC-46F2 high dose group compared to 0% observed in L19-IL2 treated group. The analysis of tumor tissues taken from mice treated with OC-46F2 as monotherapy or combination therapy confirms that the anti Syndecan-1 antibody inhibits vascular maturation and induces loss of VM structures.

These results suggest that the combined therapy could improve the therapeutic efficacy of L19-IL2 and OC-46F2 administrated as monotherapy, and that OC-46F2 may be administered at high dose without any toxic effect. On the basis of these results, it will be interesting to extend the preclinical experiments of combined therapy to a model of human melanoma cells generated from patients.

In conclusion, these findings indicate for the first time the involvement of Syndecan-1 in the process of VM in metastatic melanoma. Blocking Syndecan-1 activity by OC-46F2 antibody in combination therapy with L19-IL2 could result in potential novel therapeutic approaches for metastatic melanoma. Moreover, our data are in line with the observation that the most effective therapies should target different factors involved in the mechanisms responsible for tumor progression.

## MATERIALS AND METHODS

### Cell lines and human tissues

Cultures of primary human melanoma cells MeTA, MeMO, MeMI, MePA, MeOV, MeCoP, MeFeR, MeBO, MeTU, MeDeBo [45], human metastatic melanoma MV3 cell line from S. Ferrone (New York Medical College), human melanoma SKMEL28 (ATCC, Rockville, MD) were grown in RPMI 1640 supplemented with 10% FBS and 2% L-glutamine. The telomerase-immortalized human microvascular endothelium cell line, TIME (ATCC), was grown in Endothelial Cell Basal Medium-2 (EBM-2) which is supplied as part of the Microvascular Endothelial Cell Growth Medium-2 bullet kit (EGM-2-MV) available from Lonza/Clonetics Corporation (Basel, Switzerland). All cell lines were grown at 37°C in a 5% CO_2_ incubator. All cell lines were used within six months of resuscitation.

MeTA met were isolated from murine lung metastasis and cultured in RPMI 1640 supplemented with 10% FBS and 2% L-glutamine until 100% of cells were positive with the specific anti human Ki67 antibody and tested by immunofluorescence.

Human metastatic melanoma tissues used in immunofluorescence analyses were obtained in accordance with informed consent procedures approved by the internal Ethics Board of the National Cancer Institute (IRCCS S. Martino-IST, Italy).

### Flow cytofluorimetric analysis

For indirect one-colour cytofluorimetric analysis, cells were stained with scFv OC-46F2 (5 μg/ml) mixed with mouse monoclonal antibody immunoglobulin IgG1 anti-Myc 9E10 (2.5 μg/ml) (ATCC, Rockville, MD) or the mouse IgG1 anti-human CD31 (M0823, DAKO, Glostrup, Denmark). PE conjugated isotype specific goat anti-mouse IgG1 (Life Technologies) was used as secondary antibody. Melanoma cell lines SKMEL28 and MV3 were incubated overnight in presence or absence of 5-10 μg/ml of p-Nitrophenyl-beta-D-xylopyranoside (PNPX) (Sigma) [46]. The day after the expression of Syndecan-1 was highlighted by cytofluorimetric analysis using OC-46F2 or B-A38 (10-520-C100, Exbio, Czech Republic). Anti-CD133/1 (AC133) pure, PE-conjugated anti-c-Kit/CD117 (AC126-PE), anti-p75 neurotrophin receptor (NTR)/CD271-PE mAbs and their isotype-matched controls were purchased from Miltenyi Biotec GmbH (Bergisch Gladbach, Germany). PE-conjugated goat anti-mouse IgG1 mAb was purchased from Southern Biotechnology Associated (Birmingham, AL, USA).

### RT-PCR and qRT-PCR

Total RNA was extracted from human melanoma cells harvested using RNAeasy mini kit (Qiagen). RealMasterScript SuperMix Kit (5 Prime) was used to generate cDNA. Amplifications were performed for 30 cycles (30 s at 95°C, 30 s at 58°C, 30 sec at 72°C) for all genes except for Syndecan-1 and Nodal, performed for 35 cycles, and CD144, performed for 40 cycles using Platinum TAQ (Life Technology). PCR products were run on a 1.5% agarose gel and visualized by ethidium bromide staining. A SybrGreen-based kit (Invitrogen) was used for Real Time PCR. Primers specific for human genes are reported in Supplementary Table S2.

### Purification and characterization of human recombinant antibody OC-46F2 and immunocytokine L19-IL2

The scFv OC-46F2, the immunocytokine L19-IL2 and their controls were purified from the conditioned media of mammalian cells expressing proteins using affinity columns and characterized as previously described [22, 34]. Particularly, the OC-46F2 scFv and its control were purified on a ProteinA/Sepharose column (GE Healthcare) according to the manufacturer's instructions. The immunocytokine L19-IL2 was purified on ED-B fibronectin domain [47] conjugated to Sepharose 4B (GE Healthcare). Proteins were dialyzed against phosphate buffer saline (PBS) overnight at +4°C and sterile filtered using Millex-GP 0.22 μm filter unit (Millipore). Subsequently, they were analyzed under reducing conditions by sodium dodecyl sulfate*-*polyacrylamide gel electrophoresis (SDS-PAGE) and in native conditions by fast-protein liquid chromatography on a Superdex 200 column (Supplementary Figure S6).

### *In vitro* tubules formation assay

Glass coverslips in 24-well cell culture plates were pre-coated with reduced growth factor Matrigel at the concentration of 18 mg/ml (354263, Corning, Bedford, US) and incubated for 30 minutes at 37°C. Melanoma cells and endothelial cells were transferred at the density of 4×10^4^ or 9×10^4^ cells per well in complete specific medium, respectively, incubated at 37°C 5% CO_2_ and then were observed for their capacity to form tubule-like structures or vascular tubules.

To test the ability of scFv OC-46F2 to inhibit tubule formation by melanoma cell lines SKMEL28 transfected with empty vector pcDNA3.1 or scFv OC-46F2 or control scFv [22] were plated on Matrigel in complete specific medium and incubated at 37°C 5% CO_2_ for 48 hours. Moreover, OC-46F2 or control scFv and L19-IL2 were added at the concentration of 200 μg/ml after four to six hours when SKMEL28, MeTA and TIME started to form tubules [19]. All experiments were performed in triplicate. After fixation in 2% *paraformaldehyde* in PBS, the tubules formed were counted on ten different high magnification microscopic fields per coverslip under light microscopy (Leica Microsystems, Wetzlar, Germany) at 100X or 50X magnification for melanoma and endothelial cells, respectively. Images were captured using a DM LB2 microscope camera (Leica).

### Endothelial cell and melanoma cell co-culture on matrigel

Glass coverslips in 24-well cell culture plates were pre-coated with reduced growth factor Matrigel at the concentration of 18 mg/ml (354263) and incubated for 30 minutes at 37°C. TIME were transferred at the density of 1×10^5^cells per well in complete specific medium and incubated overnight at 37°C 5% CO_2_. The day after tubule formation, MeTA-GFP (4×10^4^cells/well) were added and real time images were analyzed with an inverted microscope. After four hours, we performed fixation in 2% *paraformaldehyde* in PBS and immunofluorescence analysis.

### *In vivo* matrigel plug assay for angiogenesis

C57BL6/J male mice (Charles River Laboratories International, Wilmington, MA, USA) were subcutaneously injected with 500 μl of reduced growth factor Matrigel phenol red free (356231, Corning) at the concentration of 10 mg/ml supplemented with 300 ng/ml FGF2 and 30UI/ml Heparin in absence or presence of 100, 200, 400 or 600 μg/mouse of scFv OC-46F2 or control scFv. After four days the animals were killed and the plugs removed [48, 49].

### Immunofluorescence and immunohistochemistry

For immunofluorescence staining, tumors or lungs were excised, embedded in cryo embedding medium (Kaltek, Italy) and stored at −80°C. Cryostat sections (6μm) of tumors were fixed in ice-cold acetone for 10 minutes and dried at room temperature. Cells were plated on glass coverslips in a 24-well cell culture plate in complete medium and were grown at 37°C in a 5% CO_2_ incubator. Cells were fixed in 2% *paraformaldehyde* in PBS for 30 minutes and permeabilized in 0,1% Triton in PBS for 10 minutes.

For immunofluorescence we used scFv OC-46F2 (at the concentration of 5μg/ml) mixed with anti-Myc (at the concentration of 1.25 μg/ml), the rat anti-mouse CD31 (clone MEC 13.3, kindly provided by A. Mantovani, Humanitas Institute, Milan, Italy), the mouse IgG1 anti-human CD31, the rabbit polyclonal anti*-*VEGF receptor-2 (ab2349, Abcam, UK), the mouse IgG2b anti human-CD144 (MAB9381, R&D Systems, Minneapolis, USA), the human L19IgG1 anti B-fibronectin [15], the goat anti human NUMA (sc-18557, Santa Cruz, Texas, USA), the chicken polyclonal anti-GFP (ab13970, Abcam), the mouse anti human ki-67 (M0722, Sigma-Aldrich), the mouse anti-human smooth muscle actin (SMA) (DAKO) and the rabbit polyclonal anti-desmin (Abcam). As secondary antibody we used the Alexa Fluor 350, 488 or 594 goat anti-mouse IgG_1_ for anti-Myc and anti-human CD31, the Alexa Fluor 594 goat anti-rat for anti-mouse CD31, the Alexa Fluor 594 goat anti rabbit for anti-VEGF receptor-2, the Alexa Fluor 594 goat anti-mouse IgG_2b_ for anti human-CD144, the Alexa Fluor 594 chicken anti goat or the Alexa Fluor 350 donkey anti goat for anti-Numa, the Alexa Fluor 488 goat anti chicken for anti-GFP and the Alexa Fluor 488 or 594 goat for L19IgG1, the Alexa Fluor 594 goat anti-mouse IgG_2a_ for anti-SMA and the Alexa Fluor 594 goat anti rabbit for anti-desmin (Life Technologies, USA). The tissue sections or cells were counterstained with DAPI using ProLong^®^ Gold Antifade Mountant (Life Technologies) or Glycer gel (DAKO). Images were captured using a ApoTome microscope with AxioCam (Karl Zeiss, Thornwood, NY, USA). The immunohistochemical procedures have been described in Orecchia et al. [22].

### Animal experimental models

Six-week-old female NOD SCID mice were originally obtained from the Charles River Laboratories International (Wilmington, MA, USA) and were bred in-house. Housing, treatment and sacrifice of animals followed national legislative provisions (Italian law no. 116, 1992 and no. 26, 2014) for the protection of animals used for scientific purposes.

Six-week-old female NOD SCID mice were subcutaneously (s.c.) injected with 5×10^6^ SKMEL28, MeTA, MeMO, MeMI, MePA, MeOV, MeCoP and MeFeR melanoma cells and the tumor growth was monitored daily. Specimens of SKMEL28 and MeTA human melanoma were obtained by subcutaneous injection of 10^7^ cells/mouse in NOD SCID mice. Animals were sacrificed when tumors reached a volume of about 1.0 cm^3^.

Six-week-old female NOD SCID mice were injected intravenously (i.v.) with the human melanoma MV3, SKMEL28, MeTA, MeMI, MePA cell lines (1.5×10^6^ cells/mouse). Eighteen days following the i.v. injection of MV3 cells and 45 days following the i.v. injection of other cell lines, mice were sacrificed and lungs were harvested.

*In vivo* treatments with purified OC-46F2 or immunocytokine L19-IL2 as monotherapy or combination therapy were performed in NOD SCID mice each s.c. injected with 10^7^ SKMEL28 human melanoma cells. When tumors were palpable, mice were randomized in the different treatment groups. *In vivo* treatments with purified OC-46F2 [22] or immunocytokine L19-IL2 [34] as monotherapy or combination therapy were performed in NOD SCID mice each s.c. injected with 10^7^ SKMEL28 human melanoma cells. When tumors were palpable different types of therapy were started.

For monotherapy, two groups of eight mice each received 80 μg scFv OC-46F2 every day from day eight to day twenty five by s.c. injection into the tail vein of each animal and 40 μg L19-IL2 every three days from day nine to day twenty one by s.c. injection into the tail vein of each animal, respectively. Similar groups of animals were untreated. For the first cycle of combined therapy with low dose (LD) or high dose (HD) OC-46F2, two groups of thirteen or twenty four mice were injected with 40 μg or 80 μg of OC-46F2, respectively, together 40 μg L19-IL2 following the same scheme of monotherapy. For the second cycle of combined therapy six mice of the LD group and three mice of the HD group were injected with 40 μg or 80 μg of OC-46F2, respectively, every day from day thirty-nine to day fifty-six day by s.c. injection, together 40 μg L19-IL2 on days forty, forty-three and forty-six into the tail vein of each animal. For the second cycle of monotherapy in the HD group we followed the same scheme for combined therapy using OC-46F2 and L19-IL2 separately. On days in which both molecules were administered, OC-46F2 was injected into the peritoneum after six hours from L19-IL2 injection into the tail vein.

Every day from tumor cell implantation, the tumor volumes were determined using the following formula: (d)^2^ x D x 0.52, where d and D are the short and long dimensions (centimeters) of the tumor, respectively, measured with a caliper. The animals’ weight was recorded daily. Animals were sacrificed when the tumor volume reached a volume between 0.8 and 1.6 cm^3^ and tumors were stored at −80°C.

### Statistical analysis

All results are presented as mean ± SEM. Statistically significance of the differences between the groups was evaluated by nonparametric Mann Whitney test using Prism 6 for MAC.

## SUPPLEMENTARY MATERIAL FIGURES AND TABLES


