# Supplementary material for: Targeting Syndecan-1, a molecule implicated in the process of vasculogenic mimicry, enhances the therapeutic efficacy of the L19-IL2 immunocytokine in human melanoma xenografts
Source: Oncotarget. 2015 Oct 9;6(35):37426–42. doi: 10.18632/oncotarget.6055 (PMC4741939; doi:10.18632/oncotarget.6055)
Supplement: Supplementary file 1 [file oncotarget-06-37426-s001.pdf]

Targeting Syndecan-1, a molecule implicated in the process of vasculogenic mimicry, enhances the therapeutic efficacy of the L19-IL2 immunocytokine in human melanoma xenografts

Supplementary Material

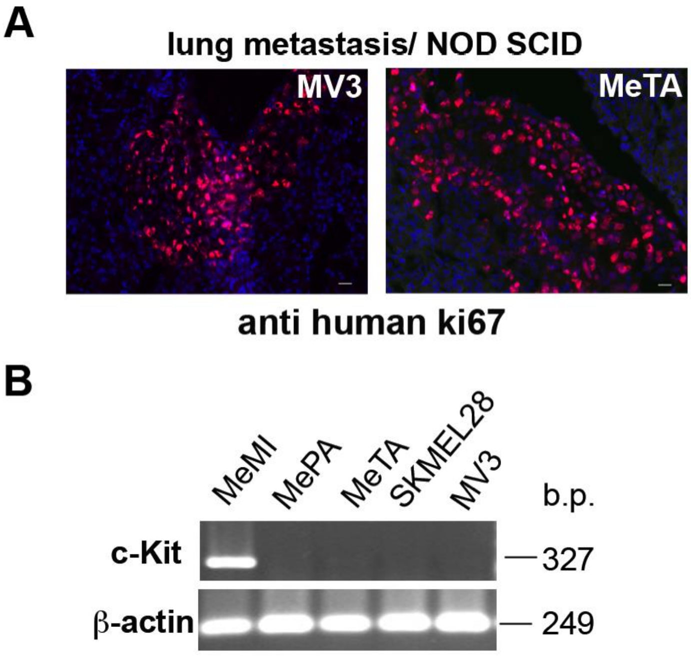

Supplementary Figure S1. Metastatic potential of melanoma cell lines. A, immunofluorescence of cryostat sections of lung metastasis, induced by MV3 or MeTA i.v. injected in NOD SCID mice, stained with anti human ki67 antibody and counterstained with DAPI. Scale bars, 50μm. B, RT-PCR analysis of c-Kit expression in MeMI, MePA, MeTA, SKMEL28 and MV3 melanoma cells. Beta-actin was used as positive control. Base pairs (b.p.) of human genes are indicated.

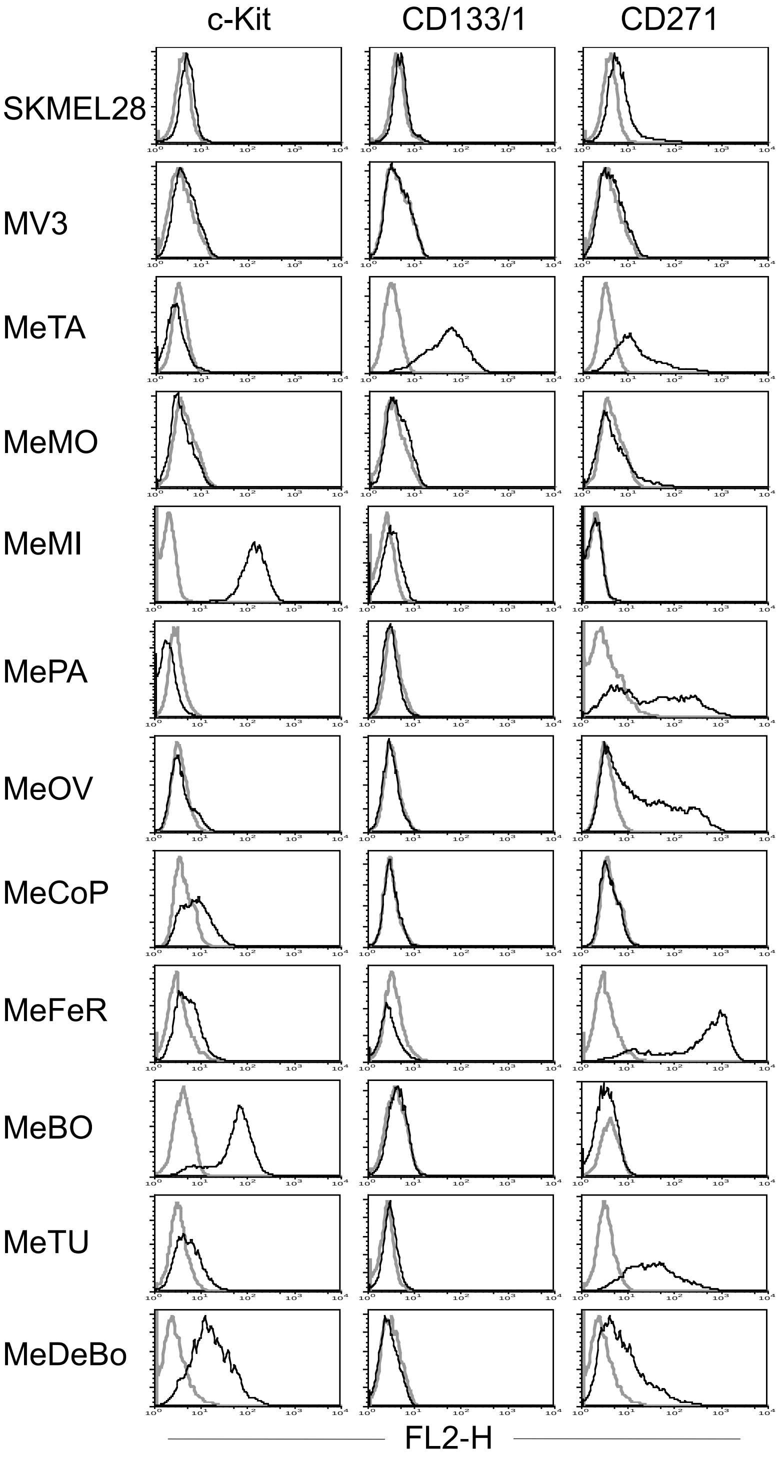

Supplementary Figure S2. Analysis of the expression of c-Kit/CD117, CD133/1 and CD271 stem cell-associated markers by melanoma cell lines. Primary mAbs to the indicated molecules and PE-conjugated goat anti mouse isotype-specific antibody as second reagent were used in Flow Cytofluorimetric analysis. Gray profiles represent negative controls.

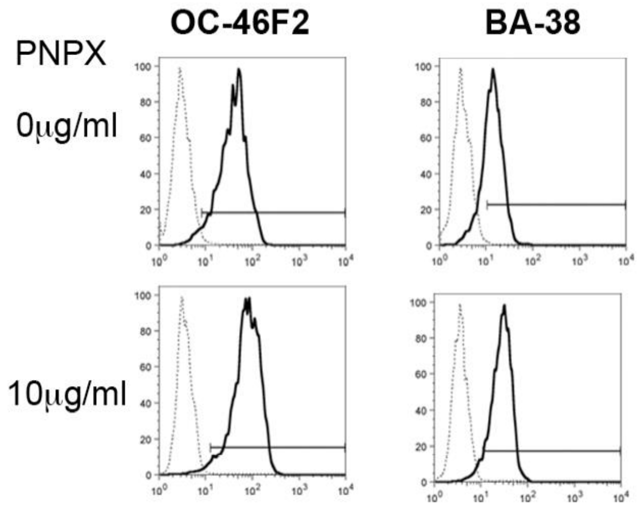

Supplementary Figure S3. scFv OC-46F2 recognizes core protein of syndecan-1. Flow cytofluorimetric analysis of human syndecan-1 expression in MV3 human melanoma cells without or with overnight treatment with 10 μg/ml PNPX, an elongation inhibitor of protein-associated GAG, using B-A38 commercial monoclonal antibody anti syndecan-1 and OC-46F2. The same results were obtained using SKMEL-28 melanoma cell line. Dashed black line represents the negative control.

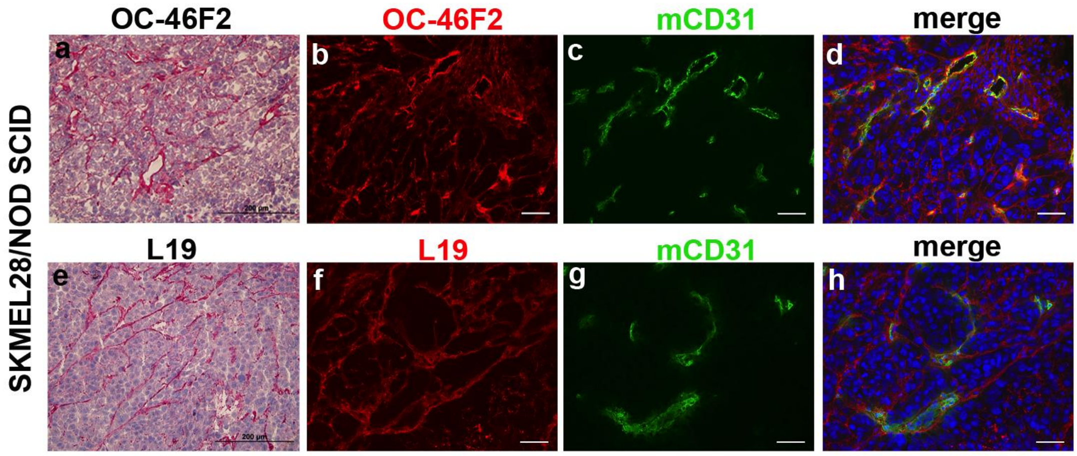

Supplementary Figure S4. Syndecan-1 and B-Fibronectin expression in melanoma tissues. Immunohistochemical (a,e) or immunofluorescence (b-d, f-h) staining of xenografted SKMEL28 human melanoma tumors grown in NOD SCID mice using anti syndecan-1, scFv OC-46F2, (a, b), anti B-FN, L19, (e, f) and anti mouse CD31 (c, g). Scale bars, 200μm (a,e) Scale bars, 50μm (b-d, f-h).

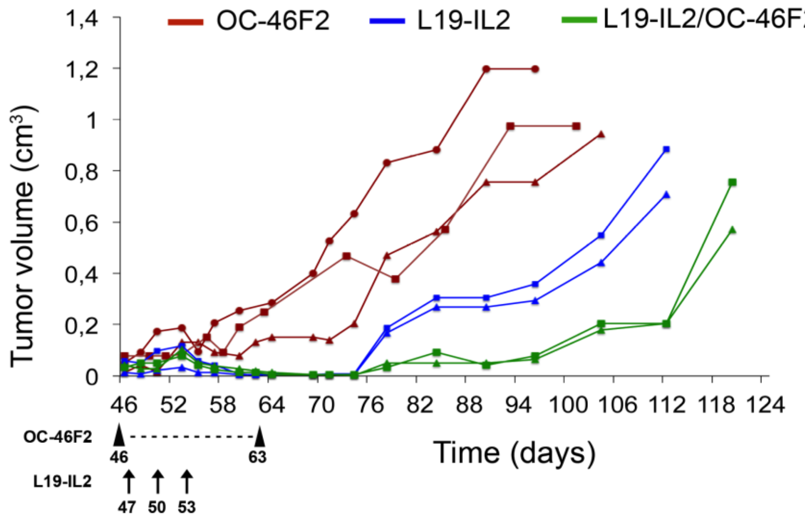

Supplementary Figure S5. Tumor growth inhibition in L19-IL2/OC-46F2 HD not responding mice. Tumor growth curves from day 46 of the L19-IL2/OC-46F2 HD not responding mice (n=7) subjected to three different treatments as indicated in the figure. Arrowheads indicate the OC-46F2 treatment administrated every day in the reported range of days. Arrows indicate the L19-IL2 treatment administrated in the reported days.

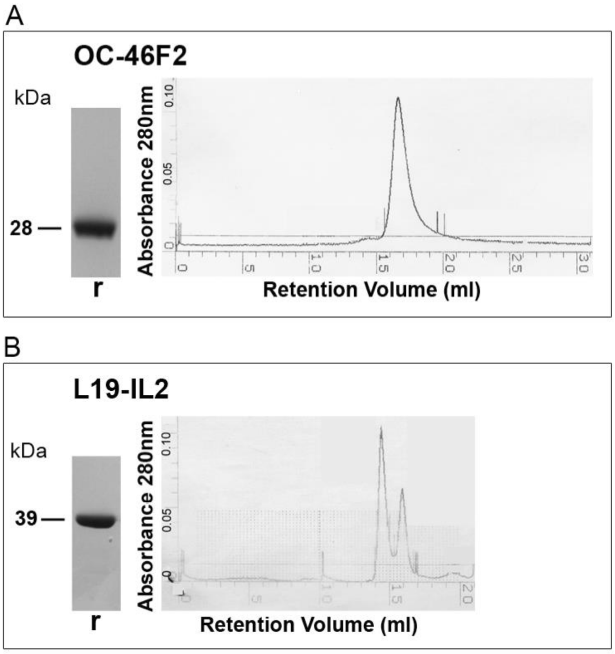

Supplementary figure S6. OC-46F2 human recombinant antibody and L19-IL2 immunocytokine proteins. SDS-PAGE analyses of the purified OC-46F2 human recombinant antibody (A, left) and L19-IL2 immunocytokine (B, left) in reducing conditions (r); the molecular masses of the proteins are reported. Size exclusion chromatography profiles (Superdex 200) of the purified OC-46F2 human recombinant antibody (A, right) and L19-IL2 immunocytokine (B, right) in native conditions.

Table S1. Protein expression of endothelial and VM markers on endothelial and metastatic melanoma cells

Protein	TIME	MeMI	MePA	MeTA	SKMEL28
CD31	+	-	-	-	-
Syndecan1	+	+	+	+	+
CD144	+	+	+	+	+
VEGFR2	+	+	+	+	+

Table S2 Primers used for RT-PCR and qRT-PCR

Human gene	Primers sequence	Size (bp)
**Syndecan-1**	Fw: 5'-tcaccttgtcacagcagacc-3'<br>Rev: 5'-gttgaggcctgatgagtggt-3'	253
**VEGFR2**	Fw: 5'-gtggggattgacttcaactg-3'<br>Rev: 5'-tgtgctgttcttcttggtca-3'	201
**CD144**	Fw: 5'-ttcatgacgtgaacgacaac-3'<br>Rev: 5'-tccaccacgatctcatacct-3'	262
**c-Kit**	Fw: 5'-tgacttacgacaggctcgtg -3'<br>Rev: 5'-aaggagtgaacagggtgtgg -3'	327
**CD44**	Fw: 5'-cctggatcaccgacagcacagaca-3'<br>Rev: 5'-ttctgacgactccttgttcacca-3'	382
**ALDH1**	Fw: 5'-ttggaatttcccgttggtta-3'<br>Rev: 5'-tgtccaagtcggcatcag-3'	352
**Nodal**	Fw: 5'-cttctccttcctgagccaacaagagg-3'<br>Rev: 5'-ggtgacctgggacaaagtgacagtg-3'	202
**beta-actin**	Fw: 5'-actccatcatgaagtgtgacg-3'<br>Rev: 5'-catactcctgcttgctgatcc-3'	249
